# A survey of public perception, knowledge and factors influencing COVID-19 vaccine acceptability in five communities in Ghana

**DOI:** 10.4314/gmj.v57i1.2

**Published:** 2023-01

**Authors:** Ernest Yorke, Maame-Boatemaa Amissah-Arthur, Vincent Boima, Ida D Dey, Vincent Ganu, Dela Fiagbe, John Tetteh, Anna Gyaban-Mensah, George Ekem-Furgurson, Alfred E Yawson, Christopher C Mate-Kole

**Affiliations:** 1 Department of Medicine & Therapeutics, University of Ghana Medical School, College of Health Sciences, University of Ghana; 2 Department of Medicine and Cardiothoracic Unit, Korle-Bu Teaching Hospital; 3 Department of Psychiatry, University of Ghana Medical School, College of Health Sciences, University of Ghana; 4 Department of Community Health, University of Ghana Medical School, College of Health Sciences, University of Ghana; 5 Department of Psychology, College of Humanities, University of Ghana; 6 Centre for Ageing Studies, College of Humanities, University of Ghana

**Keywords:** COVID-19, vaccine acceptability, perception, knowledge, community

## Abstract

**Objective:**

The present study assessed the public's perception and Knowledge about COVID-19 and factors that could affect vaccine acceptability in Ghana.

**Design:**

We carried out a cross-sectional population-based study. A structured questionnaire was used to capture data on socio-demographic information, knowledge, and the public's perception of COVID-19 infection, as well as COVID-19 vaccine acceptability from consented participants. Factors affecting vaccine acceptability in Ghana were explored. Robust ordinary least square linear regression analysis was adopted to assess factors associated with vaccine acceptability.

**Setting:**

Five communities (Labone, Lartebiorkoshie, Old Fadama, Chorkor, and Ashiyie) in Accra in the Greater Accra district were selected.

**Participants:**

WHO modified cluster-sampling method was applied to select households of 997 participants in the five communities.

**Results:**

Most respondents were males (57.6%), and the median age of participants was 30 years. The study participants demonstrated a good knowledge of COVID-19 and had high perceptions of the COVID-19 pandemic. The results revealed that the highest educational level, marital status, self-rated Knowledge of COVID-19, Knowledge of COVID-19 definition, Knowledge of COVID-19 symptoms, and perception of the COVID-19 pandemic were significantly associated with vaccine acceptability. Self-reported impact of COVID-19 lockdown/movement restrictions on agriculture and job as a source of livelihood was associated with vaccine acceptability.

**Conclusion:**

Higher subjective and objective knowledge of COVID-19 increases vaccine acceptability scores significantly thus, education on COVID-19 and the vaccination against SARS-CoV-2 infection must be intensified to improve vaccine acceptability in Ghana, especially among those with lower educational backgrounds.

**Funding:**

None declared

## Introduction

The new coronavirus infection (COVID-19) caused by the severe acute respiratory coronavirus-2 (SARS-CoV-2) strain was first identified after an outbreak in Wuhan, Hubei province, in China in December 2019. The COVID-19 pandemic has affected all continents.

The current global infection (i.e., January 12, 2021) was 89,707,115 with 1,940 352 deaths; the worst affected areas are China, Europe, the USA, Latin America, and Brazil.1 In Ghana, by February 23, 2021, the total confirmed cases of COVID-19 was 82,586, with 594 deaths.^2^ In Africa, including Ghana, most countries are experiencing a second wave of infections.^1^,^2^

As a new viral infection, many people have been exposed to mixed messaging and myths about the true nature and origins of the virus, its mode of transmission, and preventive and curative measures. The WHO and Ghana Health Service (GHS) recommend hand hygiene (hand-washing under running water and disinfection), appropriate social and physical distancing, and wearing face masks and shields.^1^,^2^ Another key intervention strategy to combat the pandemic is using the COVID-19 vaccine to achieve herd immunity and break the chain of transmission. Preliminary data for Phase 3 vaccine trials suggest 90-98% vaccine efficacy.^3^

There is a plethora of information channels and uncertainty about the authenticity of the messaging worldwide that can negatively impact global efforts to ensure mass vaccination towards achieving herd immunity. Given this, we sought to assess the public's perception and Knowledge about COVID-19 and the factors affecting vaccine acceptability in Ghana.

## Methods

### Study Design

This cross-sectional population-based study in five Greater Accra Region (GAR) communities was conducted over two months between October and November 2020.

### Study setting

Five (5) communities (Labone, Lartebiorkoshie, Old Fadama, Chorkor, and Ashiyie) in Accra in the Greater Accra region were selected. Accra is Ghana's capital and largest city, with an estimated urban population of 2,291,352 as of 2012. The Greater Accra Region (GAR) has the second largest population in Ghana (4,010 054 people) and the largest proportion of urban dwellers.^4^ Accra is divided into 11 sub-metropolitan areas. All five locations described are under the Accra Metropolitan Assembly. The neighbourhoods were selected to reflect rural, peri-urban, and urban settings in GAR with varying socio-demographic and environmental characteristics. 4

### Inclusion criteria

1. All adult residents in the communities 18 years and above

### Exclusion criteria

1. People who have already had COVID-19 infection
2. Vulnerable participants include pregnant women, children, and institutionalised persons such as incarcerated individuals.

### Sample size determination

The sample size for this study was determined using the formula for cross-sectional studies^5^:



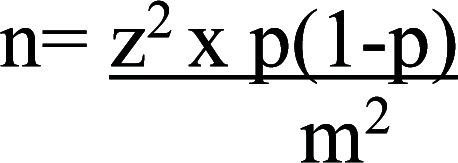



n= required sample size

z= confidence level at 95% (standard value of 1.96)

p= estimated risk perception of COVID-19 (59.0%) 6

m= margin of error at 5% (standard value of 0.05)

n= 372

Accounting for such contingencies as non-response or refusals, the sample was increased by 5%

N+ 5% of calculated sample size = 372 x 1.05 = 391.

Overall, 200 participants selected per community were recruited and interviewed, totalling 1000 participants.

### Participant Selection

The World Health Organisation (WHO) modified cluster sampling method in each of the five communities was applied to selected households.^7^ Each community was clustered using geographical or natural boundaries, and simple random sampling to chose a cluster. All adult members (≥ 18 years) in households within a chosen cluster were interviewed until the sample size was obtained. If the sample size was not obtained within a cluster, simple random sampling was used to choose the next cluster, and the process continued until the sample size was obtained.

### Data collection method

Data were collected by the investigators and trained research assistants (RAs) at each study site using structured questionnaires. The questionnaire was interpreted for the participants in a preferred local language for those who could not read or write. The RAs practised interpretation of the questionnaire in the local language during the training period. Questions from a previous publication^8^ guided the development of the questionnaire. Baseline demographic characteristics were recorded, including age, gender, ethnicity, religion, highest educational level, and occupation. The questionnaires captured data on knowledge and perception of COVID-19 infection, the self-reported impact of COVID-19 lockdown/movement restrictions on livelihoods, and COVID-19 vaccine acceptability.

### Outcome variable

The dependent variable in the study was vaccine acceptability, which was assessed by adopting a standardised attitude vaccine acceptability tool. The tool comprised 20 standard questions with 4 sub-domains (Mistrust, Con-cern, Compensation, and Altruistic).

The questions were 5-point Likert scale and the composite raw score ranged from 1-92. The focus outcome was on the raw scores; however, artificial dummy and ordinal outcomes were also generated. The dummy outcome was generated by classifying participants' scores as low (those below the overall mean score) and high (above the mean). The ordinal outcome was generated by considering the raw scores' percentile distribution. This was done by classifying participants' scores ≤25th percentile as low, 26th-74th percentile as average, and 75th and above percentile as high scores.

The questionnaire (supplement file) contained 22 items on Knowledge and perception of COVID-19 and specific questions about myths or falsehoods available at the World Health Organization's “myth busters” website.^9^ For knowledge assessment, a correct answer carried 1 mark whiles 0 for otherwise. Composite raw scores were generated for each participant and further reclassified as; scores ≤25th percentile as low, 26th-74th percentile as average, and 75th and above percentile as high scores.

### Data analysis

Data were analysed using Stata 16.1. Socio-demographic characteristics were summarised in tables. In view of the nature and normality of the outcome variables, the analysis adopted t-test and F-test statistics to assess the significant mean difference between groups. Normality test adopted the Shapiro-Wilk and Skewness and Kurtosis test for confirmation. The hypothetical idea of the study analytical procedure was assessed using three processes, first, robust least square linear regression analysis on the raw vaccine acceptability scores and estimating coefficients of linear regression equations by describing the relationship between independent variables and a dependent variable (vaccine acceptability). Poisson and ordinal logistic regression were performed independently to assess vaccine acceptability-associated factors. These analyses were performed separately on the dummy and the ordinal artificial outcomes. The significance level was set at 0.05.

### Ethical consideration

This study was conducted following the ethical code of the Helsinki Declaration on Human Experiments in 1964 (revised in 2000). The protocol was approved by the University of Ghana College of Health Sciences Ethical and Protocol Review Committee with protocol identification number of CHS-Et/M.7-5.3/2020-2021. All participants provided written consent to take part in the study.

## Results

The results of 997 respondents with complete data are presented. Most respondents were males (57.6%) aged 25 years and below (36.2%). The age ranged from 18-86 years, with 30 years median age. Over one-quarter of the participants were unemployed (27.2%) (Table 1).

**Table 1:** Demographic characteristics

Variable	Frequency N=997(%)
**Sex**	
**Male**	574(57.6)
**Female**	423(42.4)
**Age [Min-Max]**	18–86
**≤25**	370(36.2)
**26–35**	300(29.3)
**36–45**	166(16.2)
**≥46**	187(18.3)
**Age Median, IQR**	30, 18
**Ethnicity**	
**Akan**	415(41.6)
**Ewe**	172(17.6)
**Ga-Adangbe**	289(29.0)
**Other**	121(12.1)
**Religion**	
**Christianity**	910(91.3)
**Islam**	76(7.6)
**Other**	11(1.1)
**Highest Educational**	
**Primary**	329(33.0)
**Secondary**	407(40.8)
**University**	261(26.2)
**Employment**	
**Employed**	726(72.8)
**Unemployed**	271(27.2)
**Marital status**	
**Single**	542(54.4)
**Married**	359(36.0)
**Others**	96(9.6)
**Knowledge on Covid-19 definition**	
**Poor**	134(13.4)
**Low**	826(82.9)
**Average**	35(3.5)
**High**	2(0.2)
**Knowledge of Covid-19 symptoms**	
**Low**	365(36.6)
**Average**	329(33.0)
**High**	303(30.4)
**Likelihood of Covid-19 infection**	
**Unlikely**	328(32.9)
**Likely**	669(67.1)
**Wearing a face mask prevent Covid-19 infection**	
**Yes**	790(79.2)
**No**	207(20.8)
**How likely is Covid-19 a weapon**	
**Likely**	395(39.6)
**Neither**	232(23.3)
**Unlikely**	370(37.1)

In assessing the mean difference in vaccine acceptability scores between groups, the analysis revealed that the highest educational level, marital status, self-rated Knowledge of COVID-19, knowledge of the COVID-19 definition, COVID-19 symptoms, and high perception of the COVID-19 pandemic were significantly associated with vaccine acceptability (p≤0.05).

Regarding educational background, as the educational level increases, the mean score for vaccine acceptability also increases. Unmarried participants had a higher mean score of vaccine acceptability (53.21±12.26) than married participants (50.54±13.37). As Knowledge of COVID-19 increases, the vaccine acceptability score significantly increases (Table 2).

**Table 2:** Assessment of mean acceptability score by demographic characteristics

Variable	Mistrust	Concern	Compensation	Altruistic	Overall Acceptance	Test
	Mean±SD	Mean±SD	Mean±SD	Mean±SD	Mean±SD	
**Sex**						
**Male**	9.17±4.33	15.79±7.58	15.10±5.93	11.85±5.11	51.92±13.24	-0.53^t^
**Female**	8.63±4.17	16.85±7.04	15.61±5.29	11.26±4.77	52.35±12.26	
**Age**						0.27^f^
**25 and below**	9.47±4.67	17.48±7.15	15.19±5.24	12.03±4.78	54.17±12.29	
**26–35**	8.98±4.05	16.74±7.34	15.10±5.87	11.37±4.75	52.18±12.79	
**36–45**	8.51±3.91	14.35±7.08	15.57±6.59	11.21±5.28	49.64±13.62	
**46 and above**	8.24±3.97	14.69±7.55	15.71±5.29	11.46±5.37	50.1±12.59	
**Nationality**						-0.36^t^
**Ghanaian**	8.93±4.25	16.27±7.37	15.30±5.64	11.57±4.96	52.08±12.87	
**Other**	9.42±5.17	14.42±7.43	16.47±7.13	12.84±5.49	53.16±10.7	
**Ethnicity**						1.7^f^
**Akan**	8.80±4.11	16.52±7.35	15.13±5.63	11.86±4.79	52.32±12.93	
**Ewe**	9.06±4.49	17.00±7.98	16.03±5.68	11.33±5.11	53.44±13.64	
**Ga-Adangbe**	9.11±4.10	15.44±7.09	15.20±5.54	11.05±5.06	50.80±12.06	
**Other**	8.86±4.86	16.08±7.12	15.22±6.08	12.37±5.08	52.54±12.97	
**Religion**						0.68^f^
**Christianity**	8.91±4.21	16.19±7.42	15.33±5.66	11.54±4.98	51.96±12.79	
**Islam**	9.5±4.75	16.75±6.69	14.70±5.66	12.41±4.83	53.35±13.24	
**Other**	8.09±5.45	17.09±8.31	19.18±5.33	10.54±5.66	54.91±13.31	
**Highest Educational**						10.31***^f^
**Primary**	8.77±4.28	15.44±7.03	14.38±5.37	10.91±5.22	49.50±13.26	
**Secondary**	9.09±4.42	16.55±7.60	15.80±5.77	11.79±4.86	53.23±12.93	
**University**	8.92±4.01	16.75±7.37	15.77±5.76	12.17±4.73	53.61±11.60	
**Employment**						-1.25^t^
**Employed**	8.93±4.47	16.22±7.51	15.04±5.73	11.6±5.08	51.79±13.39	
**Unemployed**	8.98±3.67	16.28±7.01	16.08±5.44	11.59±4.67	52.93±11.18	
**Marital status**						4.68**^f^
**Single**	9.05±4.37	17.11±7.15	15.32±5.48	11.73±4.69	53.21±12.26	
**Married**	8.69±4.03	15.05±7.50	15.31±5.93	11.52±5.23	50.54±13.37	
**Others**	9.23±4.55	15.78±7.52	15.35±5.79	11.17±5.55	51.54±13.37	
**Self-Rated Knowledge of COVID-19**						7.66***^f^
**Poor**	8.61±4.36	15.18±7.57	14.22±644	10.04±5.16	48.04±14.87	
**Average**	8.22±4.39	16.38±7.22	15.00±5.36	11.69±4.89	52.30±12.55	
**Good**	8.73±4.09	16.40±7.49	15.99±5.69	11.97±4.92	53.10±12.25	
**Knowledge on COVID-19 definition**						14.09***^f^
**Poor**	8.81±4.29	15.13±8.24	12.44±5.71	9.62±5.23	46.01±15.51	
**Low**	8.94±4.27	16.28±7.22	15.79±5.53	11.85±4.89	52.87±12.17	
**Average**	9.31±4.17	18.94±6.42	15.11±5.69	12.97±4.13	56.34±9.25	
**High**	11.5±3.53	25.50±6.36	18.5±5.54	14.00±2.82	69.50±9.19	
**Knowledge of COVID-19 symptoms**						6.04**^f^
**Low**	8.77±4.44	16.50±8.32	14.16±5.68	10.90±5.03	50.32±13.98	
**Average**	9.16±4.15	16.30±7.13	15.51±5.84	11.67±5.13	52.66±12.63	
**High**	8.91±4.18	15.86±6.35	16.51±5.19	12.35±4.61	53.64±11.29	
**Perception about COVID-19 pandemic**						10.77***^f^
**Low**	9.59±4.85	16.31±7.44	14.75±5.79	10.92±4.95	51.57±13.86	
**Average**	8.61±3.49	15.14±6.32	15.33±5.61	11.29±4.75	50.37±11.42	
**High**	8.33±3.73	16.65±7.67	15.97±5.50	12.51±4.97	53.47±12.04	

NOTE: Superscript t and f denote the t-test and F-test statistic estimates used for mean comparison between groups. P-value Notation: **<0.01 ***<0.001

Furthermore, the impact of COVID-19 lockdown/movement restrictions on livelihoods was assessed to determine their association with attitudes toward vaccine acceptability. The lockdown/movement restrictions' impact on agriculture and job was significantly associated with vaccine acceptability.

People who were not engaged in agriculture as a source of livelihood had higher vaccine acceptability scores [No=52.30±12.73; Yes=48.91±14.06; p<0.05]. Similarly, unemployed participants had the highest vaccine acceptability score [No=53.09±12.03; Yes=50.04±14.15; p<0.001]. Although not statistically significant, selling farm produce, livestock management, trading, daily wages, and debt impacted vaccine acceptability scores (Table 3).

**Table 3:** Assessment of mean acceptability score by the self-reported impact of COVID-19 lockdown/movement restrictions on livelihood

Covid-19 lockdown/movement restrictions impact on:	Total	Mistrust	Concern	Compensation	Altruistic	Overall Acceptance	t-test
	n(%)	Mean±SD	Mean±SD	Mean±SD	Mean±SD	Mean±SD	
**Agriculture**							1.97*
**No**	938(94.1)	8.96±4.30	16.36±7.40	15.36±5.67	11.61±4.96	52.30±12.73	
**Yes**	59(5.9)	8.59±3.80	14.31±6.59	14.66±5.66	11.35±5.14	48.91±14.06	
**Selling farm produce**							1.77
**No**	921(92.4)	8.94±4.30	16.35±7.40	15.37±5.69	11.63±4.97	52.31±12.75	
**Yes**	76(7.6)	8.87±3.87	14.88±6.87	14.64±5.39	11.20±5.03	49.59±13.48	
**Livestock management**							1.87
**No**	932(93.5)	8.96±4.32	16.33±7.40	15.36±5.69	11.64±4.99	52.30±12.79	
**Yes**	65(6.5)	8.75±3.43	14.87±6.78	14.65±5.32	10.95±4.73	49.23±13.07	
**Trading**							1.18
**No**	496(49.7)	9.12±4.23	16.40±7.30	15.20±5.46	11.86±4.91	52.58±12.41	
**Yes**	501(50.2)	8.77±4.31	16.08±7.45	15.44±5.87	11.33±5.02	51.62±13.22	
**Daily wages (labourers)**							1.17
**No**	560(56.2)	8.73±4.28	16.43±7.34	15.66±5.62	11.69±5.06	52.52±12.89	
**Yes**	437(43.8)	9.20±4.24	16.00±7.42	14.88±5.71	11.47±4.86	51.56±12.74	
**Debt**							1.62
**No**	735(73.7)	8.91±4.22	16.56±7.30	15.61±5.67	11.41±4.86	52.49±12.26	
**Yes**	262(26.3)	9.03±4.42	15.35±7.50	14.51±5.60	12.09±5.26	51.00±14.28	
**Employed**							3.53**
**No**	673(67.5)	8.77±4.21	16.79±7.29	15.60±5.59	11.93±4.83	53.09±12.03	
**Yes**	324(32.5)	9.31±4.37	15.09±7.42	14.73±5.79	10.91±5.19	50.04±14.15	

NOTE: P-value Notation: *<0.05 ***<0.001

Inferential analysis assessing the association between individual factors and vaccine acceptability scores showed that age, self-rated Knowledge of COVID-19, Knowledge of COVID-19 definition and symptoms, and perception of the COVID-19 pandemic were significantly associated with vaccine acceptability. Compared with participants aged 25 years and below, those aged 36-45 had reduced vaccine acceptability by over 3-fold. However, as self-rated knowledge increases, vaccine acceptability increases.

In addition, objective Knowledge of COVID-19 definition and symptoms depicts a significantly increased score of vaccine acceptability when the objective assessment score increases by a unit score [OLS estimate: Knowledge on COVID-19 definition and symptoms β(95%CI)= 3.56(1.38 - 5.74) and 0.32[0.02 - 0.61) respectively]. An increased unit of perception of the COVID-19 pandemic score increased the vaccine acceptability coefficient by approximately 0.35(95%CI=0.13-0.57) (Table 4). Comparing results from the focus model with the binary and the ordinal outcomes, the analysis showed no different relationships (Table 4).

**Table 4:** Risk factors associated with acceptability raw scores, binary and ordinal level among participants

Variable	Focus: Raw scores	Binary	Ordinal
	OLS	Poisson	Ordered Logistic
	β[95%CI]	aPR[95%CI]	aOR[95%CI]
**Sex**			
**Male**	**Ref**	**Ref**	**Ref**
**Female**	0.68[-0.87 – 2.23]	0.99[0.80 – 1.21]	1.06[0.83 – 1.37]
**Age**			
**≤25**	**Ref**		**Ref**
**26–35**	-2.02[-4.06 – 0.02]	0.86[0.67 – 1.11]	0.76[0.55 – 1.05]
**36–45**	-3.42[-6.42 – 0.43]*	0.71[0.47 – 1.04]	0.66[0.42 – 1.03]
**≥46**	-2.57[-5.59 – 0.44]	0.76[0.51 – 1.14]	0.71[0.45 – 1.13]
**Nationality**			
**Ghanaian**	**Ref**	**Ref**	**Ref**
**Other**	1.96[-2.07 – 5.99]	1.07[0.58 – 1.98]	1.25[0.52 – 2.96]
**Religion**			
**Christianity**	**Ref**	**Ref**	**Ref**
**Islam**	2.02[-1.03 – 5.08]	1.29[0.93 – 1.79]	1.40[0.87 – 2.23]
**Other**	4.85[-2.73 – 12.4]	1.12[0.43 – 2.91]	1.38[0.45 – 4.23]
**Highest Education**			
**Primary**	**Ref**	**Ref**	**Ref**
**Secondary**	2.12[0.11 – 4.13]	1.21[0.93 – 1.59]	1.37[0.99 – 1.87]
**Tertiary**	1.53[-0.72 – 3.80]	1.05[0.77 – 1.43]	1.26[0.88 – 1.81]
**Employment status**			
**Employed**	**Ref**	**Ref**	**Ref**
**Unemployed**	-0.10[-1.77 – 1.57]	0.87[0.69 – 1.10]	0.84[0.63 – 1.12]
**Marital status**			
**Married**	**Ref**	**Ref**	**Ref**
**Single**	0.31[-2.02 – 3.65]	1.02[0.76 – 1.38]	0.99[0.70 – 1.42]
**Others**	0.83[-2.04 – 3.70]	1.11[0.76 – 1.62]	0.95[0.59 – 1.53]
**Self-Rated Knowledge of COVID-19**			
**Poor**	**Ref**	**Ref**	**Ref**
**Average**	3.19[0.49 – 5.90]*	1.06[0.75 – 1.51]	1.32[0.85 – 2.03]
**Good**	3.25[0.43 – 6.06]*	1.13[0.79 – 1362]	1.37[0.88 – 2.17]
**Knowledge on COVID-19 definition**	3.56[1.38 – 5.74]***	1.48[1.14 – 1.92]**	1.41[1.00 – 1.99]*
**Knowledge on COVID-19 symptoms**	0.32[0.02 – 0.61]*	1.02[0.98 – 1.06]	1.05[1.00 – 1.11]*
**Perception**	0.35[0.13 – 0.57]**	1.01[0.99 – 1.04]	1.02[0.99 – 1.05]

NOTE: Abbreviation; Ref=Reference category used for inferences; OLS=Ordinary Least Square Regression; aPR=Adjusted Prevalence Ratio; aOR=adjusted Odd Ration. NOTE: P-value Notation: *<0.05, **<0.01, ***<0.001

## Discussion

Our study investigated Knowledge of COVID-19, perception of COVID-19, and factors associated with vaccine acceptability among Ghanaians. The study participants demonstrated fairly good Knowledge of COVID-19 and had highly positive perceptions of covid-19. Findings from the study revealed that higher educational level, marital status, self-rated Knowledge of COVID-19, Knowledge of COVID-19 definition, Knowledge of COVID-19 symptoms, and perception of COVID-19 pandemic significantly determined vaccine acceptability. Age, self-rated Knowledge of COVID-19, Knowledge of COVID-19 definition and symptoms and perception of the COVID-19 pandemic were significant factors associated with vaccine acceptability.

About 85% of respondents knew COVID-19 as a respiratory disease caused by a viral infection. This was slightly higher than that reported by another study.^10^ Close to 96% of respondents knew COVID-19 was associated with respiratory symptoms accompanied by fever and was contagious and hence could be easily transmitted from one person to the other. This finding is similar to that reported by another study in China.^11^ Surprisingly, most respondents (98.5%) did not believe that the novel coronavirus could lead to death though it could progress to severe illness.

Regarding knowledge of symptoms of COVID-19, most participants correctly identified fever (70.5%) and cough (85.8%) as a symptom of COVID-19. This is not surprising since these are some of the most commonly reported symptoms associated with COVID-19.^12^ Quite surprisingly, between 52-90% of respondents indicated that shortness of breath/difficulty in breathing, sore throat, loss of smell and taste, fatigue/tiredness, diarrhoea and runny or stuffy nose were not symptoms of COVID-19. Another study reported that about 18% of participants had partial knowledge of COVID-19 symptoms, which is far lower than our findings.^10^

Most participants were very concerned about this outbreak (72.5%). This supports findings from another study which indicated that participants were concerned about the outbreak and the potential risk of infection of any member of their families.^13^ Many respondents believed that the government was not exaggerating the threat (59.9%), and 73.0% believed the government could effectively handle the crisis, whilst only 26.7% agreed that health officials could effectively handle the crisis. These results are consistent with findings by Lim and colleagues, who reported that most participants found the communication from government officials trustworthy and associated with a high likelihood of adopting protective behaviour.

Thus, government policies and communication to the public on COVID and its prevention will be vital in decreasing the spread and fatality of this infection.^14^ Also, other studies reported that higher levels of trust in government sources of information are more likely to lead to the acceptability of a vaccine.^15^,^16^ In contrast, Oleribe and colleagues found that government institutions' responses to the pandemic in Nigeria were rated poor, with the federal president's office receiving the poorest rating.^17^ This emphasises the need to enhance public communication on the disease and vaccine deployment to improve public confidence in the ability of health officials to respond adequately to the pandemic.

The overall vaccine acceptance increased as the educational level increased. Mistrust of the government officials and information provided on COVID-19, as well as non-compliance with COVID-19 preventive measures, decreased as the educational level increased. These findings were similar to a study in the United States which found that as years of education increase, so does report acceptance of the COVID-19 vaccine.^18^ Likewise, vaccine refusal and hesitancy among the working-age population in France were significantly associated with a lower educational level.^19^ Also, COVID-19 vaccine attitudes such as social concern and altruistic vaccination among participants increased as the educational level increased. These results contradict other studies in US and UK, where people with higher educational attainment were less likely to accept vaccination.^20^

The participants' marital status significantly determined vaccine acceptability, with single people having higher mean acceptability than married participants. Similar results were obtained for the various vaccine acceptance attitudes: mistrust, social concern, risk compensation, and altruistic vaccination.

It is worth noting that better self-rated Knowledge of COVID-19 increased mean vaccine acceptability scores. Again, an increase in Knowledge of COVID-19 symptoms and better mean scores of the definition of COVID-19 increase vaccine acceptability. Inferential analyses confirmed the above factors as significant predictors of vaccine acceptability among the participants.

Findings from our study revealed that the impact of COVID-19 on agriculture and employment was significantly associated with vaccine acceptability. People not engaged in agriculture as a source of livelihood had a higher vaccine acceptability score than those engaged in agriculture. This could be attributed to the fact that participants who engage in agriculture perceive a lower risk of contracting the virus because they interact less with the general public and spend most of their time outdoors/in open spaces. Unemployed participants had the highest vaccine acceptability scores. This finding is similar to a study among dental professionals in Israel which found a significant positive correlation between the unemployment rate and willingness to receive a COVID-19 vaccine.^21^ Carson and colleagues on COVID-19 vaccine hesitancy and acceptability in multi-ethnic communities found employment as a barrier to vaccination.

Factors accounting for the low acceptability among employed people were concerns about taking time off work or obtaining sick leave to attend vaccination.^22^ On the contrary, Malik et al. reported that unemployed participants had lower COVID-19 vaccine acceptance when compared to those employed or retired in the United States.^18^

The present study was conducted among a Ghanaian population in the Greater Accra region, which involved participants in rural, peri-urban, and urban areas to reflect the socio-demographic distribution of the country. Although very few participants were above 60 years, they are vulnerable to COVID-19-associated complications and must be targeted for vaccination. Thus, it is very important to target this group in future studies to ascertain their true perception, knowledge and willingness to accept the COVID-19 vaccine.

## Conclusion

The study revealed that most Ghanaians have good knowledge and perceptions about COVID-19. Age, highest educational level, marital status, objective and subjective knowledge, and perception of COVID-19 were significantly associated with vaccine acceptability. The self-reported impact of covid-19 on livelihoods such as agriculture and jobs was associated with vaccine acceptability. More effort is needed to educate the vast majority of Ghanaians with lower educational backgrounds to improve vaccine acceptability and successful implementation of vaccination programmes. There is a crucial need for transparent and consistent communication by government officials and clear support from health officials in building public confidence in the COVID-19 vaccination programme.

## Appendix 1 Descriptive characteristics of Knowledge on COVID-19 definition and symptoms among participants

**Table:**

Variable	Response		Total
	No	Yes	
	n(%)	n(%)	n
***Knowledge on Coronavirus definition***			
Novel coronavirus is a respiratory disease caused by a viral infection	155(15.5)	842(84.5)	997
Symptoms usually include respiratory symptoms accompanied by fever, but novel coronavirus is not contagious	952(95.5)	45(4.5)	997
Novel coronavirus can progress to a severe illness but never leads to death	982(98.5)	15(1.5)	997
***Symptoms of the novel coronavirus***			
Fever	294(29.5)	703(70.5)	997
Cough	142(14.2)	855(85.8)	997
Shortness of breath/difficulty in breathing	519(52.1)	478(47.9)	997
Sore throat	683(68.5)	314(31.5)	997
Runny or stuffy nose	578(58.0)	419(42.0)	997
Muscle or body aches	833(83.5)	164(16.5)	997
Headaches	558(56.0)	439(44.0)	997
Fatigue (tiredness)	750(75.2)	247(24.8)	997
Diarrhoea	894(89.7)	103(10.3)	997
Constipation	38(3.8)	959(96.2)	997
Loss of smell and taste	807(80.9)	190(19.1)	997

## Appendix 2 Descriptive characteristics of COVID-19 perception among participants

**Table:**

Perception variable	SD	D	N	A	SA	DK
I am very concerned about this outbreak	38(3.8)	33(3.3)	22(2.2)	175(17.5)	725(72.7)	4(0.4)
I believe that the government is exaggerating the threat.	355(35.6)	242(24.3)	45(4.5)	110(11.0)	157(15.7)	88(8.8)
I trust that health officials can effectively handle this crisis.	90(9.0)	82(8.2)	67(6.7)	354(35.5)	372(37.3)	32(3.2)
I trust that the government can effectively handle this crisis	88(8.8)	94(9.4)	61(6.1)	368(36.9)	360(36.1)	26(2.6)
I expect this outbreak to get larger	344(34.5)	352(35.8)	59(5.9)	129(12.9)	179(17.9)	34(3.4)

SD- Strongly disagree, D- Disagree, N-Neutral, A-Agree, SA-Strongly Agree, DK-Don't Know

## Appendix 3 Descriptive characteristics assessing the COVID-19 attitude scale for vaccine acceptability

**Table:**

Domain	Level of response					
	Strongly Disagree	Disagree	Neutral	Agree	Strongly Agree	Total
***Mistrust***	n(%)	n(%)	n(%)	n(%)	n(%)	n
There is a cure for COVID-19 but govt is keeping it	487(55.4)	269(30.6)	28(3.2)	44(5.0)	51(5.8)	879
COVID-19 is a man-made virus that was created to get rid of c certain groups of people	327(37.3)	215(24.5)	57(6.5)	102(11.6)	176(20.1)	877
Sometimes I think the government is using COVID-19 to kill off people who are not wanted by society	530(56.3)	304(32.3)	19(2.0)	41(4.4)	47(5.0)	941
The government already has a COVID-19 vaccine but is keeping it from the public	480(54.7)	303(34.5)	20(2.3)	29(3.3)	45(5.1)	877
Pharmaceutical companies cannot be trusted to produce COVID-19 Vaccine since it would cut into their drug profits	304(35.4)	238(27.7)	85(9.9)	116(13.5)	116(13.5)	859
***Concern***						
I would be concerned about how my family might react to my getting a COVID-19 vaccine	250(26.6)	211(22.4)	26(2.8)	182(19.4)	271(28.8)	940
I would be concerned about how my partner/friend might react to my getting a COVID-19 vaccine	279(29.6)	201(21.4)	22(2.3)	200(21.3)	239(25.4)	941
I would be concerned about confidentiality (others finding out) if I received COVID-19 vaccine	279(29.5)	222(23.5)	56(5.9)	153(16.2)	235(24.9)	945
I would be concerned that getting COVID-19 vaccine would affect my ability to get health insurance	288(32.9)	265(30.3)	47(5.4)	111(12.7)	164(18.7)	875
I would be concerned that getting COVID-19 vaccine would lead	266(28.1)	235(24.9)	35(3.7)	150(15.9)	259(27.4)	945
It concerns me that if I get COVID-19 vaccine the covid 19 a	177(20.2)	151(17.3)	47(5.4)	141(16.1)	359(41.0)	875
***Compensation***						
Getting COVID-19 vaccine means you can interact with people in person without wearing facemask	131(13.5)	112(11.5)	20(2.1)	327(33.7)	381(39.2)	971
COVID-19 vaccine will make wearing of facemask less important	244(25.1)	197(20.2)	42(4.3)	270(27.7)	221(22.7)	974
If I get COVID-19 vaccine I will be more likely to move around without wearing a facemask, social distancing or washing my hands regularly	169(17.3)	109(11.1)	25(2.6)	287(29.3)	389(39.7)	979
COVID-19 will no longer be a threat when the	322(33.6)	220(22.9)	69(7.2)	164(17.1)	184(19.2)	959
Everyone who gets COVID-19 vaccine will be protected against Covid-19	223(23.7)	287(30.5)	93(9.9)	174(18.5)	165(17.5)	942
***Altruistic***						
I would get COVID-19 vaccine even if I thought the vaccine might not protect me 100% against Covid-19 infection	305(31.0)	171(17.4)	28(2.8)	194(19.7)	285(29.0)	983
I would get COVID-19 vaccine that would prevent me from being able to infect other people with Covid-19, even if the vaccine might not protect me against Covid-19	352(36.0)	171(17.5)	36(3.7)	196(20.0)	224(22.9)	979
I would be one of the first people to get COVID-19 vaccine	430(44.2)	200(20.6)	40(4.1)	109(11.2)	194(19.9)	973
My willingness to get COVID-19 vaccine is important for the good of all people	132(13.7)	105(10.9)	74(7.7)	245(25.5)	405(42.1)	961
